# Curative effect of Omeprazole under different treatment courses in treatment of children with PU and HP infection and its influence on inflammatory factors

**DOI:** 10.12669/pjms.36.7.3048

**Published:** 2020

**Authors:** Shaohui Zhang, Yuan-da Zhang, Qing-wei Dong, Fang Gu

**Affiliations:** 1 Shaohui Zhang, Department of Pediatric Medicine, Baoding Children’s Hospital, Baoding 071000, Baoding, China; 2 Yuan-da Zhang, Department of Pediatric Medicine, Baoding Children’s Hospital, Baoding 071000, Baoding, China Department of Pediatric Medicine, Baoding City Children Respiratory and Digestive Diseases Clinical Research Key Laboratory, Baoding 071000, Baoding, China; 3 Qing-wei Dong, Department of Pediatric Medicine, Baoding Children’s Hospital, Baoding 071000, Baoding, China; 4 Fang Gu, Department of Pediatric Medicine, Baoding Children’s Hospital, Baoding 071000, Baoding, China

**Keywords:** Helicobacter pylori, Omeprazole, Peptic ulcer, Treatment course

## Abstract

**Objective:**

To compare curative effect and safety of omeprazole under different treatment courses in treatment of children with peptic ulcer (PU, diameter≤1.0cm) and helicobacter pylori (HP) infection and its influence on inflammatory cytokines.

**Methods:**

The study was a randomized controlled study and conducted at Baoding children’s hospital from June 2015 to June 2018. In this study 100 PU children with positive HP were chosen and classified into two groups at random. The 58 cases in the observation group were given omeprazole + amoxicillin + clarithromycin, and the antibiotics were not used two weeks later. Then, omeprazole was used to treat for two weeks. 42 cases in the control group were given omeprazole + amoxicillin + clarithromycin for two weeks. Curative effect, HP eradication rate, clinical symptoms, incidence of adverse reactions, level of serum inflammatory cytokine interleukin-6 (IL-6) and level of tumor necrosis factor-a (TNF-a) in two groups were compared.

**Results:**

Total effective rate, HP eradication rate and clinical symptom relief of observation group were better than those of control group, and the differences showed statistical significance (P>0.05). The differences of two groups in the incidence of adverse reactions had no statistical significance (P>0.05). Serum IL-6 level and TNF-a level of observation group were significantly lower than those of control group and before the treatment, and the differences had statistical significance (P>0.05).

**Conclusion:**

The application of omeprazole in treatment of PU patients with positive HP for four weeks can significantly improve PU cure rate and HP eradication rate, relieve clinical symptoms and reduce inflammatory response, so it deserves to be promoted clinically.

## INTRODUCTION

Peptic ulcer (PU) is one of the common digestive system diseases in children. The incidence of this disease presents the rising trend in China.^1^ Due to children’s special physiological and immune characteristics, the incidence of this disease is high. With the universal use of pediatric gastroscope and other examination means, the detection rate of small peptic ulcer in children increases. The focus of small peptic ulcer (diameter ≤1.0cm) is most common in PU children, accounting for about 70%.^2^ PU in children most happens to stomach and duodenum, showing periodic and rhythmic epigastric pain.^3^ HP infection is a major pathogenic factor of PU and chronic gastritis in children.^4^ Through the investigation, HP infection rate for children is 10%~80%.^5^

At present, the conventional triple therapy proton pump inhibitor (PPI) + amoxicillin + clarithromycin are mostly adopted clinically. This scheme can fast improve symptoms and avoid repeated relapse.^6^ With such scheme, HP eradication rate is about 70%~90%.^7^ However, the misuse of antibacterial drugs results in gradual increase of drug resistance, and the eradication function of conventional triple therapy will gradually weaken.^8^ Fourteen days therapy of “standard triple therapy” is mostly adopted in China,^9^ while US recommends 10 days or 14 days treatment.^10^ The curative effect and safety of omeprazole with different course treatment in treatment of PU children are rarely reported. In this study, the curative effect of omeprazole with different course treatment in treatment of PU children with positive HP was explored.

## METHODS

### Baseline data

100 PU (diameter≤1.0cm) children with positive HP which were diagnosed at Baoding children’s hospital from June 2015 to June 2018 were chosen and classified into two groups at random. There were 58 cases in the observation group, and 42 cases in the control group. The comparison differences of patients in gender, age, course of disease and disease type had no statistical significance (P>0.05), as shown in Table-I.

**Table-I:** Baseline data comparison of 2 groups (n/%) (X±s).

Group	No.	Gender		Age	Course of disease (year)	Disease type		
	
		Male	Female			Gastric ulcer	Duodenal ulcer	Compound ulcer
Observation group	58	32	26	12.53±2.27	1.26±0.34	19(32.76)	30(51.72)	9(15.52)
Control group	42	31	11	12.43±2.11	1.18±0.36	16(38.10)	22(52.38)	4(9.52)
t/X^2^		0.020		0.273	0.237	1.231	0.020	1.591
P		0.887		0.772	0.813	0.221	0.886	0.208

### Ethical approval

The study was a randomized controlled study and approved by the Institutional Ethics Committee of Baoding Children’s Hospital, and written informed consent was obtained from all participants

### Inclusion criteria

1. Diagnosed with PU by gastroscope, single gastric ulcer/duodenal ulcer, average diameter ≤1.0cm.
2. HP positive.
3. Did not receive systemic therapy or take acid-inhibitory drug, antibiotics and gastric mucosa protectant within 1 month before the treatment.^11^,^12^
4. Age 6~16 years old.

### Exclusion criteria

1. Stomach/duodenum showed malformation or the operation was performed.
2. Congenital internal secretion and genetic metabolic disease.
3. Gastric ulcer/duodenal ulcer were compound ulcer and ulcer like frost-spot.
4. Those with poor medication compliance could not cooperate to complete the research.

### Diagnostic criteria

PU was diagnosed as per *Diagnostic Criteria for Chronic Gastritis and Peptic Ulcer in Children by Gastroscopy* formulated in 2003.^13^ HP infection was diagnosed by positive rapid urease test (RUT) of gastric mucosal tissue or positive 13C urea breath test (13C-UBT) as per *Expert Consensus on Diagnosis and Treatment of Helicobacter Pylori Infection in Children*.

### Therapeutic method

The observation group was given omeprazole + amoxicillin + clarithromycin, and the antibiotics were not used 2 weeks later. Then, omeprazole was used to treat for two weeks. The control group was given omeprazole + amoxicillin + clarithromycin for two weeks. Drug dose: omeprazole 0.8mg/(Kg.d), taken at a draught in the morning; amoxicillin clavulanate 50mg/(Kg.d), clarithromycin 20 mg/(Kg.d), twice/d, taken orally.

### Observation indexes

Gastroscope reexamination was conducted four weeks later to confirm anabrosis recovery after the course of treatment was finished; 13C-UBT or RUT was reexamined to confirm HP eradication. If it is negative, HP eradication succeeds.

### (2) Curative effect criteria:

1. ***Recovery:*** digestive tract symptom disappears, ulcer surface disappears and scar forms.
2. ***Effective:*** digestive tract symptom is relieved, and ulcer area shrinks at least 50% compared with pretreatment.
3. ***Ineffective:*** digestive tract symptom is not relieved, and ulcer area does not shrink obviously and even expands.
4. The improvement of epigastric pain, sour regurgitation and belching was compared. Each symptom is scored 0-3. The higher the score, the more severe clinical symptoms.^14^
5. Adverse reactions such as emesis, nausea, dizziness and rash were compared.
6. Five milliliter fasting venous blood was drawn before and after the treatment, and put still for 20 minutes. Then, it was centrifuged at1500r· min^-1^ for five minutes, and the serum was taken. ELISA was used to detect the concentration of IL-6 and TNF-a.

### Statistical method

SPSS22.0 statistical software was used for data analysis. Enumeration data were expressed with rate (%), and tested with x^2^. Measurement data were expressed with x±s. Independent-sample T test was adopted for inter-group comparison, and paired-T test was applied for intra-group comparison. Chi square test was used to compare the two sample rates. P<0.05 means the difference has statistical significance.

## RESULTS

Total effective rate and HP eradication rate of observation group were obviously higher than those of control group, and the differences had statistical significance (P>0.05), as shown in Table-II.

**Table-II:** Comparison of curative effect and HP eradication rate (n/%)

Group	No.	Recovery	Effective	Ineffective	Total effective rate	Hp eradication rate
Observation group	58	49(84.48)	7(12.07)	2(3.45)	56 (96.55)	55(94.83)
Control group	42	25(59.52)	9(21.43)	8(19.05)	34(80.95)	32(76.19)
X^2^					12.187	14.021
P					<0.001	<0.001

Clinical symptoms of observation group such as epigastric distention, epigastric pain, sour regurgitation and belching were lower than those of control group and before the treatment, and the differences had statistical significance (P>0.05), as shown Table-III.

**Table-III:** Comparison of clinical symptoms (X±s).

Group	No.	Epigastric distention		Epigastric pain		Sour regurgitation		Belching	

		Before treatment	After treatment	Before treatment	After treatment	Before treatment	After treatment	Before treatment	After treatment
Observation group	58	2.61±0.26	0.81±0.09*#	2.45±0.36	0.63±0.12*#	2.18±0.33	0.63±0.07*#	2.06±0.24	0.64±0.06*#
Control group	42	2.68±0.25	1.40±0.15*	2.44±0.34	1.29±0.23*	2.16±0.32	1.31±0.17*	2.08±0.27	1.17±0.14*
t		1.356	23.610	0.139	17.499	0.450	26.609	0.559	24.357
P		>0.05	<0.05	>0.05	<0.05	>0.05	<0.05	>0.05	<0.05

*Note:* comparison before and after treatment: P*<0.05; comparison with the control group: P#<0.05

The comparison differences of both groups in adverse reactions such as emesis, nausea, dizziness and rash had no statistical significance (P>0.05), as shown in Table-IV. Serum IL-6 level and TNF-a level of observation group were significantly lower than those of control group and before the treatment, and the differences had statistical significance (P>0.05), as shown in Table-V.

**Table-IV:** Comparison of adverse reactions (n%).

Group	No.	Emesis	Nausea	Dizziness	Rash	Total
Observation group	58	3(5.17)	3(5.17)	2(3.45)	3(5.17)	55(94.83)
Control group	42	2(4.76)	3(7.14)	3(7.14)	3(7.14)	32(76.19)
X^2^						8.682
P						>0.05

**Table-V:** Comparison of inflammatory cytokine concentration (X±s).

Group	No.	IL-6 (ng/l)		TNF-a((μg/l)	

		Before treatment	After treatment	Before treatment	After treatment
Observation group	58	31.13±10.26	24.93±6.29*#	44.74±11.30	33.93±8.28*#
Control group	42	31.02±10.37	21.36±6.31*	43.94±10.61	29.67±6.38*
t		1.850	3.321	1.161	8.710
P		P>0.05	P<0.05	P>0.05	P<0.01

*Note:* comparison before and after treatment: P*<0.05; comparison with the control group: P#<0.05

## DISCUSSION

Peptic ulcer is a chronic inflammation of gastric mucosa caused by different causes, and it is the first place in the incidence of gastrointestinal diseases.^15^ The main manifestation is recurrent abdominal pain. It is usually aggravated after eating, the pain site is not exact, and it is mostly in the umbilical region, and some children are characterized by loss of appetite, fatigue, and the pain area can be more extensive in the middle upper abdomen or umbilical region.^16^ It seriously affects the quality of life of the patients. Most of the children show umbilical fullness, pain, and even vomiting in severe cases, which seriously affects the quality of life of children, and due to the poor resistance of children, it will also affect their immune function. Therefore, early treatment is of great significance to children. The occurrence factors of peptic ulcer are complex, including HP infection, excessive gastric acid secretion, infection, systemic diseases, poor diet and living habits, emotional stress, heredity, etc.^17^-^19^ When the local inflammatory response of patients is prolonged, the inflammatory response of TNF-a will be enhanced, and even the local inflammatory response will be enhanced. Therefore, the serum levels of IL-6 and TNF-a are closely related to the changes of peptic ulcer.^20^,^21^

In this study, the treatment plan of omeprazole + clarithromycin + amoxicillin was chosen. Omeprazole is a proton pump inhibitor, which is easy to concentrate in acidic environment. Through disulfide bond, omeprazole can irreversibly combine with the sulfhydryl group of proton pump, thus inhibiting proton pump of gastric parietal cells, ultimately effectively inhibiting gastric acid secretion, reducing the damage to gastric mucosa, increasing the concentration of antibiotics and improving the white blood cell function of patients. The reason is that omeprazole has a strong inhibitory effect on the basic gastric acid and gastric acid secretion caused by stimulation, which can improve the gastric acid base, reduce the stimulation of gastric acid on gastric mucosa, and finally reduce the level of inflammatory factors. Immune function refers to the body’s resistance to diseases. Usually, the immune system plays its role according to immune recognition. It is the interaction of lymphocytes, monocytes and other related cells and their products. Clarithromycin is a new generation of macrolide drugs, with strong bactericidal activity for HP and good stability in acid environment. It can inhibit protein synthesis and play an antibacterial role through hindering 50S subunit connection in cell nucleus. So, it is the most effective drug for HP.^22^ Amoxicillin is the only β-lactam antibiotics to eliminate HP, can promote ulcer surface healing and effectively control bleeding. But its anti-HP action depends on low gastric acid environment.^23^

At present, there is still no uniform standard about the treatment course of PU in children. It takes at least four weeks to treat duodenal ulcer in adults, and 6-8 weeks for gastric ulcer.^24^ PU in children has the features of acute onset, simple medical history, small ulcer area, shallow surface, strong regenerative capacity of mucous membrane and strong repair ability. In this study, PU children with positive HP were given different treatment courses of omeprazole.

### Limitations of this study

In this study, we did not carry out stratified study according to the severity of peptic ulcer. In the next step, we will study the course of drug treatment according to the severity of peptic ulcer.

## CONCLUSION

The addition of omeprazole in treatment of PU patients with positive HP for four weeks can significantly improve PU cure rate and HP eradication rate, relieve clinical symptoms and reduce inflammatory response, so it deserves to be promoted clinically.

### Authors’ Contributions:

**SZ** **and YDZ** designed this study and prepared this manuscript, and are responsible and accountable for the accuracy or integrity of the work.

**QWD and FG** collected and analyzed clinical data.

**LPH and** **YZ** significantly revised this manuscript.
